# Disease management of patients with immune thrombocytopenia—results of a representative retrospective survey in Germany

**DOI:** 10.1007/s00277-020-04173-5

**Published:** 2020-07-25

**Authors:** Anne Sophie Kubasch, Jens Kisro, Jörg Heßling, Holger Schulz, Hans-Jürgen Hurtz, Martine Klausmann, Achim Ehrnsperger, Claudia Willy, Uwe Platzbecker

**Affiliations:** 1 Department of Hematology, Cellular Therapy and Hemostaseology, University Hospital Leipzig, Leipzig, Germany; 2 Lübecker onkologische Schwerpunktpraxis, Lübeck, Germany; 3 Onkologie am Segelfliegerdamm, Berlin, Germany; 4 Praxis internistischer Onkologie und Hämatologie, Frechen, Germany; 5 Onkologische Gemeinschaftspraxis und Tagesklinik, Halle, Germany; 6 Gemeinschaftspraxis Dr. Klausmann, Aschaffenburg, Germany; 7 Novartis Pharma GmbH, Nürnberg, Germany

**Keywords:** Immune thrombocytopenia, Treatment landscape, Platelet count, Germany, Survey

## Abstract

Clinical research has resulted in an improvement of treatment options for patients with immune thrombocytopenia (ITP) over the last years. However, only few data exist on the real-life management of patients with ITP. To expand the knowledge, a multicenter, national survey was undertaken in 26 hematology practices distributed all over Germany. All patients with a diagnosis of ITP were documented using questionnaires, irrespective of the diagnosis date over a period of 2 years. Overall, data of 1023 patients were evaluated with 56% of patients being older than 60 years. Seventy-nine percent of the patients had chronic (> 12 months), 16% persistent (> 3–12 months), and 5% newly diagnosed (0–3 months) ITP. In 61% of cases, the disease lasted 3 or more years before survey documentation started. Main strategies applied as first-line therapy consisted of steroids in 45% and a “watch and wait” approach in 41% of patients. During second- and third-line strategies, treatment with steroids decreased (36% and 28%, respectively), while treatment modalities such as TPO-RAs increased (19% and 26%, respectively). As expected, patients with a low platelet count and thus a higher risk for bleeding and mortality received treatment (esp. steroids) more frequently during first line than those with a higher platelet count. Up to a third of patients were treated with steroids for more than a year. Overall, our study provides a cross-section overview about the current therapeutic treatment landscape in German ITP patients. The results will help to improve therapeutic management of ITP patients.

**Electronic supplementary material:**

The online version of this article (10.1007/s00277-020-04173-5) contains supplementary material, which is available to authorized users.

## Introduction

Immune thrombocytopenia (ITP) is an autoimmune disorder, characterized by transient or persistent decrease of the platelet count (less than 100 × 10^9^/l) [1–3]. The incidence is estimated to be 1.9–6.4/100,000 children per year and 3.3/100,000 adults per year [4]. Often, patients with ITP have no clinical symptoms and the diagnosis is due to a routine blood analysis. In other cases, ITP is diagnosed because of mild bleeding symptoms, with severe bleeding being a relatively rare event [5]. The diagnosis of ITP is established only after exclusion of secondary causes of thrombocytopenia, like infections, autoimmune or myeloid disorders, as there are no diagnostic tests to confirm ITP [3].

The main goal of current therapeutic strategy is to prevent bleeding. While the 2011 guidelines of the American Society of Hematology (ASH) suggest treatment for newly diagnosed adult patients with a platelet count < 30 × 10^9^/l irrespective of bleeding symptoms [3], there is little clinical evidence in support of this strategy using a stringent platelet cutoff. Therefore, the current German guidelines recommend that treatment decisions should be based on relevant bleeding symptoms (World Health Organization grades 3 and 4), individual bleeding risk profile, and current disease stage irrespective of platelet count. Patients with a low platelet count (20–30 × 10^9^/l) can be offered treatment, but in case of no bleeding symptoms, a “watch and wait” strategy is also an option. Thus, individual treatment decisions in ITP patients are taking into account various factors such as disease and bleeding history, age of patient, side effects of therapy, patient preference, and others [6].

Due to a better understanding of the disease pathology, treatment options for patients with ITP have increased over the last years. Steroids are still the standard first-line treatment for newly diagnosed ITP patients. In cases where a rapid increase of platelet count is required (e.g., severe bleeding, before surgery), intravenous immunoglobulins (IVIG) are usually administered in addition to steroids. Before the introduction of thrombopoietin receptor agonists (TPO-RAs), splenectomy and rituximab have been possible second-line treatment options, even though rituximab is not approved for treatment of ITP. TPO-RAs act by stimulating platelet production, proliferation, and differentiation of megakaryocytes in the bone marrow. Currently, two TPO-RAs have been approved within the EU: eltrombopag and romiplostim [5, 6]. The current German guidelines state that TPO-RAs can be offered as second-line treatment for patients with chronic ITP and studies have shown that they are effective in patients, irrespective of splenectomy status [7] or age [8]. Patients are usually responding to TPO-RAs treatment within 2 weeks, but until now, no predictive biomarkers for response are known. Even though TPO-RAs are not approved for first-line treatment, their use in this setting can be taken into consideration to treat patients with life-threatening bleeding [6]. As third-line treatment, other immunosuppressive agents (e g., azathioprine and ciclosporin) can be considered.

To date, only few data exist on the real-life management of patients with ITP in Germany. A retrospective analysis published in 2016 evaluated 422 patients who were diagnosed with ITP between 06/1995 and 12/2014. It was shown that most ITP patients can be managed as outpatients by experienced hematologists and that the current therapy guidelines were followed [9].

To expand the knowledge about real-life management of patients with ITP in Germany, a multicenter, national survey was undertaken during the years of 2016 and 2017, describing the diagnostic and treatment patterns of patients with ITP managed by hematologists in routine care in Germany.

## Materials and methods

A retrospective data collection using questionnaires was performed by 26 hematology practices distributed all over Germany. From 02/2016 to 12/2017, all patients with a diagnosis of ITP were documented, irrespective of the diagnosis date. To provide a representative patient cohort, all available data from patients with a diagnosis of ITP and a platelet count ≤ 100 × 10^9^/l were included.

The 26 participating hematology practices provided all available data of their ITP patients by means of a questionnaire specifically designed for the retrospective data collection. The following data (among others) of patient characteristics and history were collected: sex, age, disease manifestation at diagnosis, stage and classification, diagnostic workup, and platelet count. The questionnaire also included parameters concerning the course of the disease and included (among others) type of therapy and duration of therapy (for the complete questionnaire, see supplemental material).

Next to the overall evaluation, patients were additionally grouped by platelet count at diagnosis and selected parameters (reasons for diagnosis, therapeutic strategies, duration of treatment) were compared between four groups: (1) 0–10 × 10^9^/l, (2) 11–30 × 10^9^/l, (3) 31–50 × 10^9^/l, and (4) 51–100 × 10^9^/l, to distinguish possible treatment differences between patients with lower and higher platelet counts.

Data analysis was performed with Microsoft Office Business 365 Excel 2016. No statistical analysis was performed, and results are descriptive only.

The datasets generated and/or analyzed during the current study are available from the corresponding author on reasonable request.

## Results

### Patients

Within this multicenter (26 hematology practices), national survey during the years of 2016 and 2017, the data of 1023 patients were retrospectively evaluated. At diagnosis, 56% patients were > 60 years old. Fifty-three percent were female and 47% male. Seventy-two percent suffered from primary ITP, 15% from secondary ITP, and 13% were not classified. Underlying causes for secondary ITP were drug-related (27%), autoimmune disorders (23%), infections (15%), as well as other (28%) and unknown (7%) causes. Seventy-nine percent of all patients had chronic, 16% persistent, and 5% newly diagnosed ITP. In 61% of cases, the disease lasted 3 or more years before survey documentation started. Incidental findings were the main reason that led to the diagnosis of ITP (41%). At time of diagnosis, 49% of patients had a platelet count above 50 × 10^9^/l. Only 12% of patients had a very low platelet count of 0–10 × 10^9^/l. A bone marrow biopsy was performed in 50% of evaluated patients to exclude other hematologic diseases. There was a slight difference in the frequency of bone marrow biopsies according to age of patient (supplemental figure 1). Patient characteristics are shown in Table 1.

**Table 1:** Patient characteristics

Sex, *n* (%)	
Female	541 (52.9)
Male	481 (47.1)
Age, *n* (%)	
0–6	0 (0)
7–17	4 (0.4)
18–30	104 (10.,2)
31–50	174 (17.0)
51–60	171 (16.7)
> 60	570 (55.7)
ITP classification, *n* (%)	
Primary	735 (72.1)
Secondary	150 (14.7)
Not classified	134 (13.2)
ITP stage, *n* (%)	
Newly diagnosed	48 (5.1)
Persistent	154 (16.2)
Chronic	748 (78.7)
Disease manifestation at diagnosis, *n*^a^ (%)	
Gastrointestinal bleeding	19 (1.5)
Vaginal bleeding	37 (3.0)
Nosebleed	65 (5.2)
Other bleedings	80 (6.4)
Petechiae	172 (13.7)
Hematomas	242 (19.3)
Incidental finding	514 (41.1)
Unknown	123 (9.8)
Platelet count (in × 10^9^/l) at time of diagnosis, *n* (%)	
0–10	123 (12.0)
11–30	192 (18.8)
31–50	211 (20.7)
51–100	495 (48.5)
Bone marrow biopsy	
Yes	511 (50.0)
No	492 (48.2)
Unknown	18 (1.8)

^a^Multiple answers allowed (in this case, *n* equals the number of answers given and not number of patients)

### Disease manifestation at diagnosis according to platelet count

**Fig. 1:**
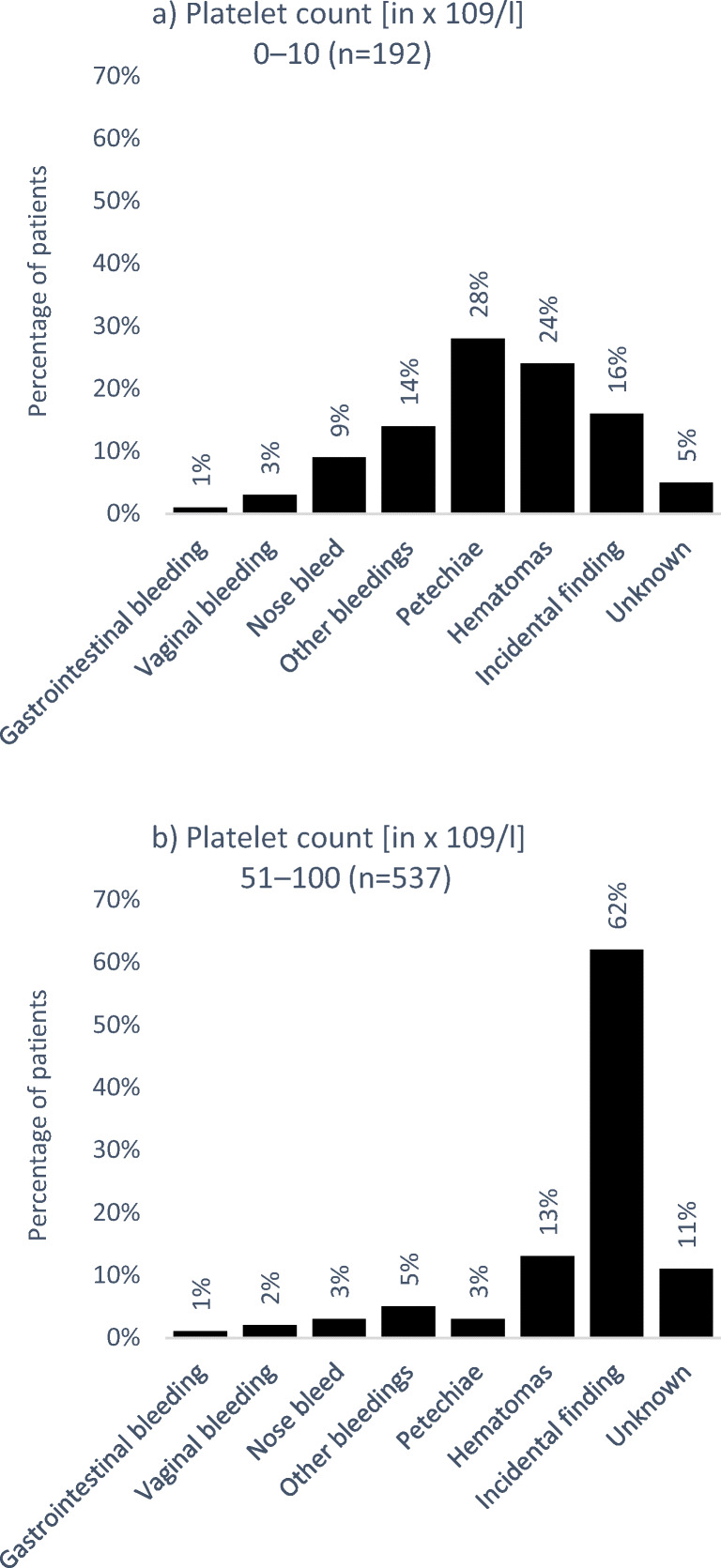
Disease manifestation at diagnosis according to platelet count at diagnosis in patients with platelet count of **a** 0–10 or **b** 51–100. Multiple answers allowed (in this case, *n* equals the number of answers given and not number of patients; percentages indicate main answer given and not the proportion of patients)

The symptoms occurring at diagnosis of ITP differed between the groups. In patients with the lowest platelet count (0–10 × 10^9^/l), the occurrence of petechiae and hematomas was the main reasons for diagnosis of ITP (52%; Fig. 1a). This was similar for patients with a platelet count of 11–30 × 10^9^/l (53%). While those two disease manifestations were frequently present at diagnosis of ITP in patients with a platelet count of 31–50 × 10^9^/l (35%), in the same percentage of patients, the diagnosis was due to an incidental finding (35%). This percentage increased in patients with a platelet count of 51–100 × 10^9^/l, with as many as 62% of diagnoses being made due to incidental findings (Fig. 1b). For figures of patients with a platelet count of 11–30 as well as 31–50 × 10^9^/l, see supplemental figure 2.

### Therapeutic strategies

The main strategies applied as first-line therapy consisted of steroids in 45% and a “watch and wait” approach in 41% of patients, followed by IVIG in 7% and platelet concentrates in 3%. Second-line therapy consisted of steroids in 36%, TPO-RAs in 19%, IVIG in 18%, “watch and wait” in 14%, rituximab in 5%, splenectomy in 3%, as well as platelet concentrates, chemotherapy, and others in 2% each. Third-line therapy consisted of steroids in 28%, TPO-RAs in 26%, “watch and wait” in 13%, IVIG in 11%, rituximab in 7%, splenectomy as well as platelet concentrates in 4% each, chemotherapy in 1%, and other therapeutic strategies in 6%. At the time of the survey, 62% of evaluated patients were ‘free of therapy’.

### Treatment decision based on platelet count

**Fig. 2:**
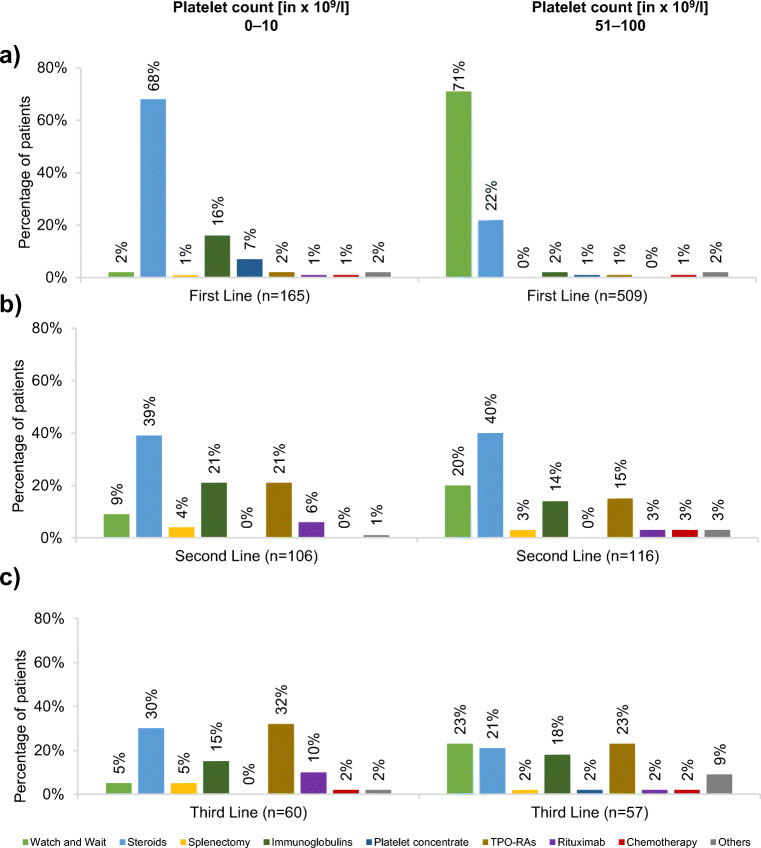
Treatment strategies according to platelet count during **a** first-, **b** second-, and **c** third-line treatment. Multiple answers allowed (in this case, *n* equals the number of answers given and not number of patients; percentages indicate main answer given and not the proportion of patients receiving treatment)

Therapeutic strategies differed according to the patient’s platelet count at diagnosis. In patients with a platelet count of 0–10 × 10^9^/l, the main strategy applied as first-line therapy were steroids (68%) followed by IVIG (16%) and platelet concentrates (7%), see Fig. 2a (left panel). During second- and third-line treatment, respectively, IVIG (21% and 15%), TPO-RAs (21% and 32%), and also rituximab (6% and 10%; off-label) were increasingly used; however, steroids (39% and 30%) were still one of the main treatment modalities (Fig. 2b and c; left panel).

**Fig. 3:**
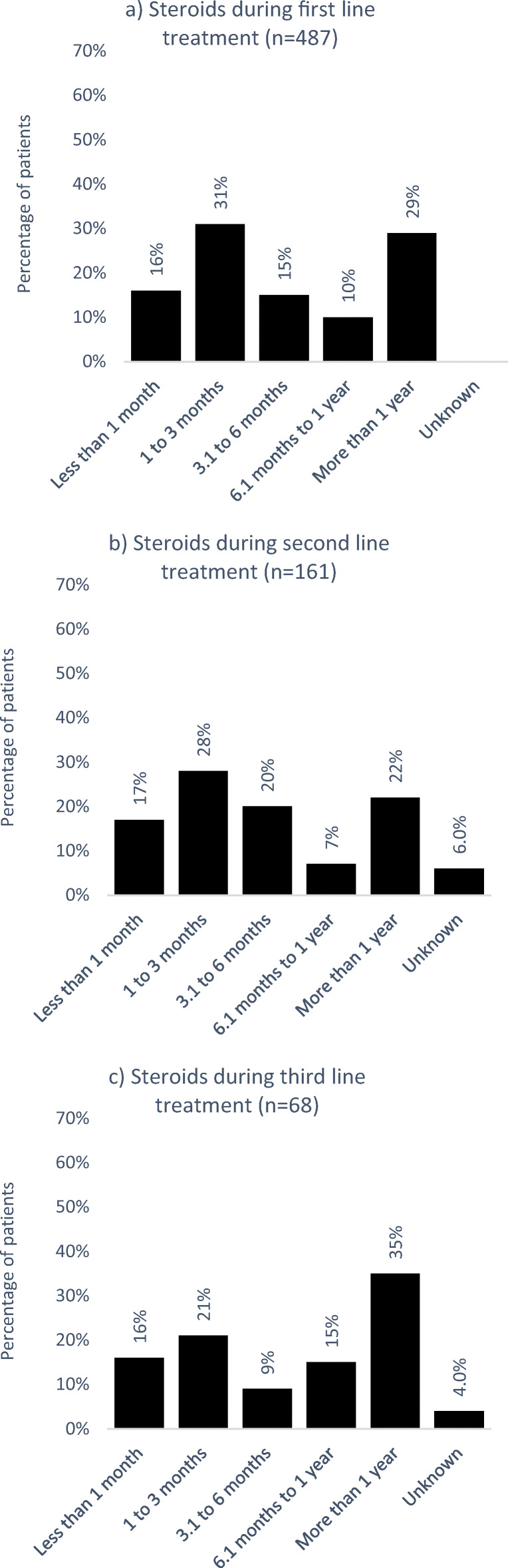
Duration of treatment with steroids during **a** first-, **b** second-, and **c** third-line treatment

A similar treatment pattern was observed in patients with a platelet count of 11–30 × 10^9^/l, where steroids were also the most commonly used first-line strategy (68%) and the use of TPO-RAs increased with further treatment lines (first line: 2%, second line: 21%, third line: 29%; see supplement material figure 3a–c; left panel). While steroids were still the main strategy applied during first line in patients with a platelet count of 31–50 × 10^9^/l (53%), followed by a “watch and wait” approach (36%; see supplemental figure 3a–c; right panel), 71% of patients in the group with the highest platelet count (51–100 × 10^9^/l) did not receive medical treatment during first-line therapy (“watch and wait”). During second and third line, respectively, multiple treatments were given, especially steroids (40% and 21%), IVIG (14% and 18%), and TPO-RAs (15% and 23%), a “watch and wait” approach was also common (20% and 23%; Fig. 2a–c, right panel). Dosage and type of steroids were not assessed during this retrospective study.

### Treatment decision based on age

Therapeutic strategies differed slightly depending on the patient’s age. While younger (≤ 60 years) and older patients (> 60 years) were treated similarly during first- and second-line therapeutic courses, younger patients (≤ 60 years) more often received rituximab (12%) or splenectomy (7%) as third-line therapy compared to older patients above 60 years of age (3% and 2%, respectively; see supplemental figure 4).

### Splenectomy

Overall, 5% of patients underwent splenectomy. Splenectomy was conducted mainly during second- (3% of patients) or third-line therapy (4% of patients) while only 1% of patients underwent splenectomy during first-line therapy. In 46% of the patients undergoing splenectomy, the operation was carried out within 12 months of diagnosis.

The frequency of splenectomy was dependent on the platelet count of the ITP patients. Patients in the two groups with the higher platelet counts (31–50 × 10^9^/l and 51–100 × 10^9^/l) underwent splenectomy only in 5% and 2% of cases, respectively, for patients with lower platelet counts (11–30 × 10^9^/l and 0–10 × 10^9^/l), splenectomy was a treatment choice for 9% and 11%, respectively.

### Duration of steroid treatment

As observed, steroids are the main treatment option during first line. Sixty-two percent of patients treated with steroids during first-line treatment received these for a duration of up to 6 months. Twenty-nine percent of the patients treated with steroids received them for more than a year, and this was still seen during second- and third-line treatment (22% and 35%, respectively; Fig. 3a–c).

### Duration of TPO-RA treatment

**Fig. 4:**
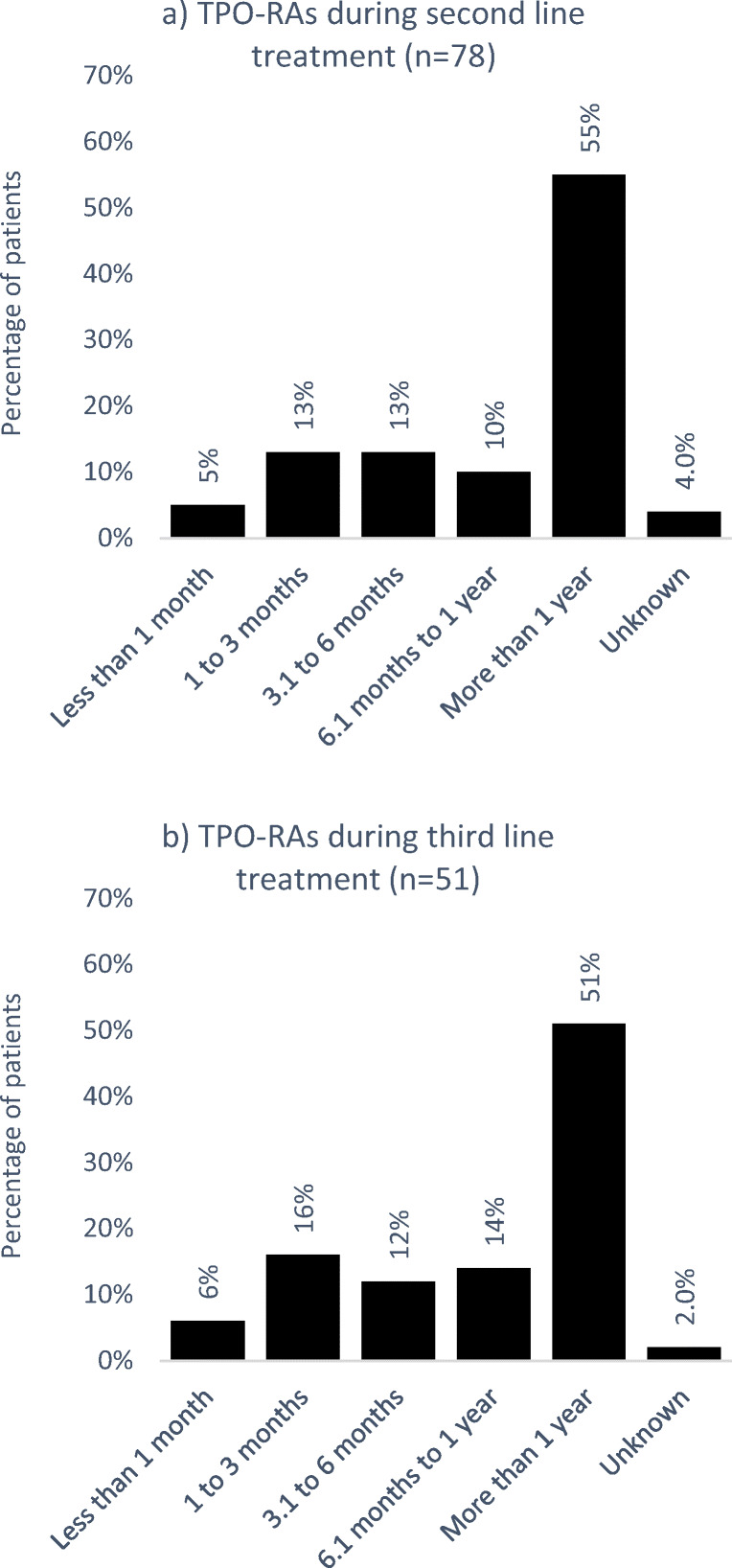
Duration of treatment with TPO-RAs during **a** second- and **b** third-line treatment

Nineteen percent of patients received TPO-RAs during second line and 55% of the patients were treated for more than a year (Fig. 4a). During third line, 51% of the patients received TPO-RAs or more than a year (Fig. 4b)

## Discussion

This study reflects the treatment reality of patients with ITP in Germany. Overall, the data of 1023 patients treated in 26 hematology practices distributed all over Germany were evaluated, giving a strong picture of real-world ITP treatment. Our study cohort represents a typical ITP population, with the majority of patients diagnosed with primary ITP (72%). In most cases, incidental findings were the main reason for diagnosis of ITP. For patients with very low platelet count (0–10 × 10^9^/l), ITP was more often diagnosed because of mild bleeding symptoms with petechiae and hematomas being the most frequent.

The treatment options most frequently used were steroids, immunoglobulins, and TPO-RAs. Rituximab was only used infrequently, and also splenectomy was seldom carried out. Interestingly, the last two treatment options (rituximab, splenectomy) were administered more frequently in younger patients (≤ 60 years of age) compared to the older patient group (> 60 years). Our findings are consistent with the recently reported results of an Italian study by Palandri et al., where age of ITP patients also influenced therapeutic decision making [10].

During first-line therapy, most patients either received steroids or no therapy (“watch and wait”), which is in accordance with current German guidelines [6]. As expected, patients with a low platelet count and thus a higher risk for bleeding and mortality received treatment (esp. steroids) more frequently during first line than those with a higher platelet count. Steroids were also the main treatment option during second-line therapy. This tendency to administer a second course of steroids before switching to an alternative therapy may be due to the use of dexamethasone in case of first-line prednisone failure, which was also observed in a retrospective study from Italy analyzing changes in therapeutic choices over a time period of 35 years [11]. Unfortunately, we did not differentiate between different types of steroids and dosing regimens in our survey. The comparatively lower application rate of TPO-RAs during second line may be due to the fact that romiplostim and eltrombopag were only approved in Germany in 2009/2010. In our survey, all patients with a diagnosis of ITP were included in the data analysis irrespective of diagnosis date, making it likely that historical patient data were included from a time period where treatment with TPO-RAs was not yet approved or included in guideline recommendations. Indeed, in approximately a third of the included patients, time since initial diagnosis was more than 5 years. This could explain the more widespread use of steroids in second-line treatment in comparison to TPO-RAs. To test this hypothesis, further studies are needed comparing treatment of patients before and after second-line approval of TPO-RAs and analyzing the treatment changes over time. Furthermore, our study cohort included 15% of patients with secondary ITP, a group for which TPO-RAs are not approved, another reason why the application of TPO-RA was less frequent than expected.

Steroids are used to recover platelet count quickly and are not intended for long-term therapy due to severe side effects. A long-term treatment period does not improve response rate and should be avoided due to significant adverse events such as infections, high blood sugar, weight gain, and osteoporosis [6]. Despite these known risks, the duration of steroid treatment is often too long [12], and to a large extent, this was also seen in the patients of our study. Nearly a third of the patients treated with steroids received them for more than a year during first-line treatment and this high percentage of patients was carried forward in the subsequent lines. These results indicate a need for initiatives aimed at restricting the use of steroids in Germany and, when appropriate, encouraging the use of other long-term treatment options like TPO-RAs for patients with ITP.

For second-line therapy, splenectomy has been considered the treatment of choice for many years. However, splenectomy is limited by the risk of surgical complications and a possibly increased risk of thrombosis and infectious complications in the long term [13]. With the introduction of medical alternatives such as TPO-RAs, the use of splenectomy has declined and is generally reserved for steroid-refractory patients with profound thrombocytopenia and high bleeding risk. Over the last three decades, splenectomy has therefore shifted from second- to third-line therapy [11]. In our data set, splenectomy was ultimately used in 5% of all patients, a relatively low number compared to other studies where splenectomy rates were around 15–21% [9, 11]. However, both of these studies were monocentric and may reflect center-specific operation rates. For our study, we included data from 26 hematology practices and splenectomy rates ranged from 0 to 19% between different practices, thereby possibly explaining those study differences.

The approval of TPO-RAs has provided more therapy choices for chronic ITP management and has broadened the ITP treatment landscape. Clinical studies have shown promising results for both eltrombopag and romiplostim. In our study, we did not differentiate between the two agents; however, while head-to-head studies between the two agents are missing, indirect comparisons suggest that there are no significant differences between eltrombopag and romiplostim regarding efficacy and safety [14, 15]. Within our study cohort, only about 20% of patients received TPO-RAs. As discussed above, this may be due to the fact that for some patients starting second-line treatment, TPO-RAs had not been approved yet. While approval was granted in 2009/2010, up until 2015, treatment was only indicated for splenectomized patients with chronic ITP (third line) or for patients in second line with a contraindication for splenectomy. The treatment pattern in our study cohort reflects this approval status, with more patients receiving TPO-RA treatment during third line in comparison to second-line treatment. Furthermore, despite the term “chronic ITP” (lasting more than 12 months [3]) only being defined after the market launch of both TPO-RA agents and even though the pivotal clinical studies included patients with a shorter disease duration, TPO-RA treatment of patients with a disease duration of less than 12 months was formally not approved. These barriers could have further discouraged the use of TPO-RAs in our study population. Only recently has this been changed for eltrombopag, which is now indicated for patients with primary ITP lasting 6 months or longer from diagnosis and who are refractory to other treatments [16]. Retrospective studies have shown that the use of TPO-RAs in the treatment of patients with newly diagnosed ITP in daily clinical practice is as effective and safe as it is in chronic ITP [17, 18] and an ongoing clinical trial is currently evaluating this line of treatment in children (NCT03939637). Therefore, more changes in clinical treatment methods are to be expected in the future and further real-world studies are needed to depict the changing clinical treatment landscape.

Strengths of our study include its relatively large sample size and the geographically distributed recruitment, which ensures the representativeness of our sample. However, the present study also has some limitations. The main limitations of our study are the retrospective design and the method of a survey in itself. We cannot rule out the possibility of some bias in data collection and patients’ selection. To minimize bias, all available data from patients with a diagnosis of ITP and a platelet count ≤ 100 × 10^9^/l were included without selection by in- or exclusion criteria. While this approach minimized bias and increased patient numbers, false diagnoses of ITP cannot be ruled out. Furthermore, due to the inflexible nature of a survey, our results may not be as valid as results obtained using methods of more comprehensive data collection. Data errors due to non-responses may exist, thus creating bias in our sample. Additionally, answer options may have led to unclear data as they may be interpreted differently by respondents. Nonetheless, our study describes the therapeutic strategies used in a German cohort of patients with ITP which sometimes do not fit the current treatment recommendations. These results can help to improve therapeutic management of ITP patients in Germany.

## Electronic supplementary material

ESM 1 (DOCX 1192 kb)

## Data Availability

The datasets generated during and/or analyzed during the current study are available from the corresponding author on reasonable request.
