# Supplementary material for: Disease management of patients with immune thrombocytopenia—results of a representative retrospective survey in Germany
Source: Ann Hematol. 2020 Jul 25;99(9):2085–93. doi: 10.1007/s00277-020-04173-5 (PMC7419449; doi:10.1007/s00277-020-04173-5)
Supplement: Supplementary file 1 — (DOCX 1192 kb) [file 277_2020_4173_MOESM1_ESM.docx]

**Supplemental material**

**Questionnaire**

1. **General data**
   1. Age in years
   2. Sex
   3. Time since initial diagnosis
   4. What were the reasons that led to the diagnosis of ITP?
   5. How was ITP classified?
   6. In case of secondary ITP, what was the reason?
   7. What stage is the patient at?
   8. Were coagulation parameters tested (prothrombin time, aPTT)?
   9. Was a differential blood count carried out?
   10. Was a bone marrow biopsy performed to exclude other hematologic diseases?
   11. How high was the platelet count (× 10^9^/l) at diagnosis?
   12. Was a splenectomy carried out?
   13. If so, how much time passed between diagnosis and splenectomy?
   14. Did the patient become refractory after splenectomy?
   15. If so, how much time passed between splenectomy and refractory status?
   16. Were further therapies necessary after failure of splenectomy?
   17. How many appointments did the patient attend at your center within the last year?
   18. How many consultations did the patient attend within the last year?
   19. Last contact with the patient was XX months ago.
2. **Information about the current clinical situation and therapy**
   1. How high was the platelet count (× 10^9^/l) at the last visit?
   2. If the patient is > 60 years and also has a platelet count ≤ 50 x 10^9^/l, can a subjectively increased bleeding tendency be noted?
   3. Is the leucocyte count currently within the reference range?
   4. Is the erythrocyte count currently within the reference range?
   5. What is the current bleeding score (according to WHO)?
   6. Is the patient currently enrolled in a study?
   7. Which therapies were given during first line?
   8. How long were first-line therapies given (except splenectomy)?
   9. Was freedom of therapy achieved after first-line therapy?
   10. If the patient became refractory after first line, how much time did pass?
   11. Which therapies were given during second line?
   12. How long were second-line therapies given (except splenectomy)?
   13. Was freedom of therapy achieved after second-line therapy?
   14. If the patient became refractory after second line, how much time did pass?
   15. Which therapies were given during third line?
   16. How long were third-line therapies given (except splenectomy)?
   17. Was freedom of therapy achieved after third-line therapy?
   18. If the patient became refractory after third line, how much time did pass?
   19. What is the current therapy line of the patient?
   20. Which therapies are currently given?
   21. Is the patient currently free of therapy?
   22. If the patient is free of therapy, through which therapy was this achieved?
   23. If a TPO-RA is currently administered, how is the TPO-RA administered?
   24. If a TPO-RA is currently administered, is the dosage dependent on body weight?

**Figures**

**
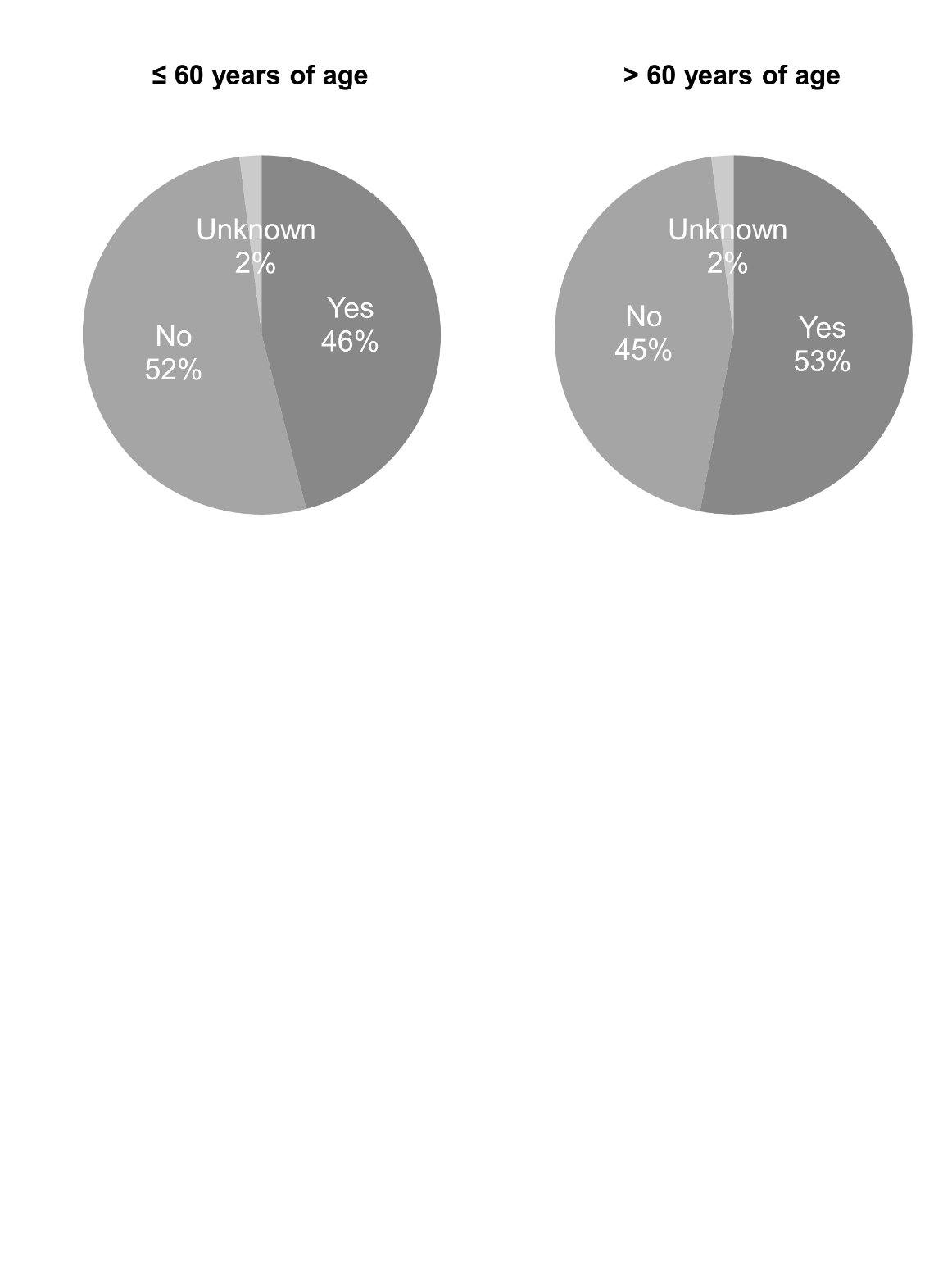
**

**Supplemental Fig. 1 Bone marrow evaluation according to age at diagnosis.**

**
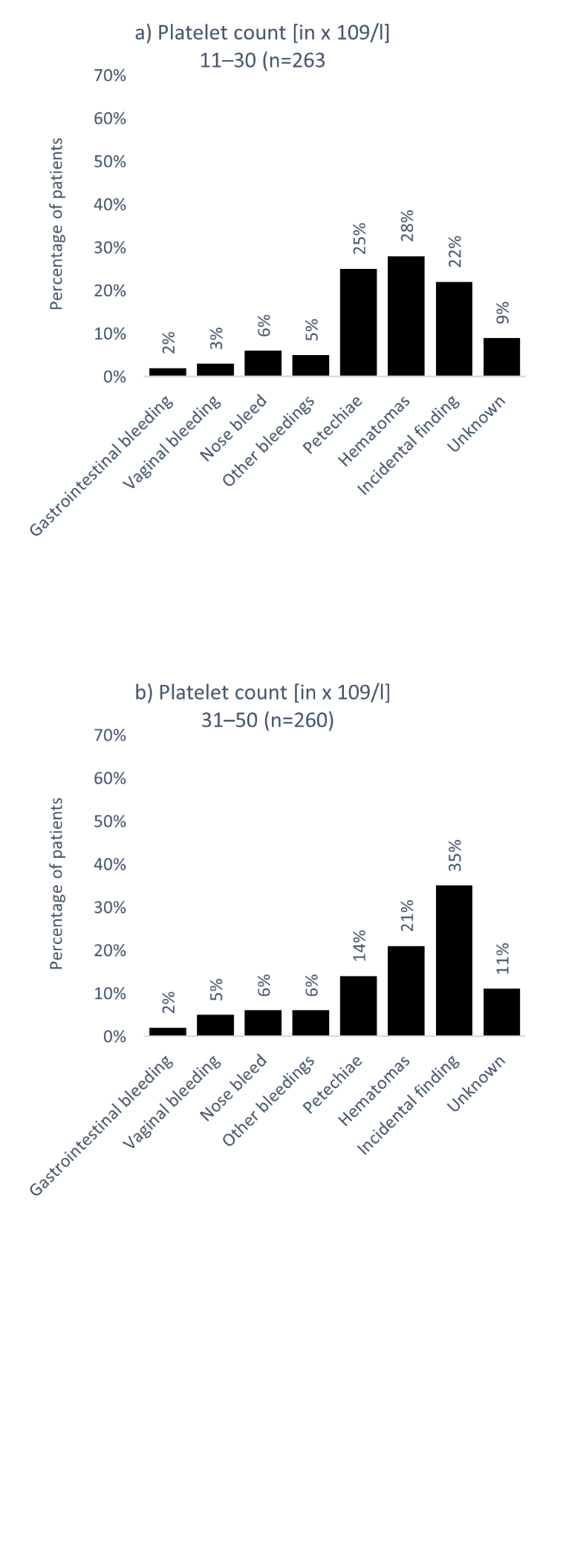
**

**Supplemental Fig. 2 Reasons for diagnosis according to platelet count at diagnosis in patients with platelet count between a) 11 and 30 or b) 31 and 50. Multiple answers allowed (in this case n equals the number of answers given and not number of patients; percentages indicate main answer given and not the proportion of patients).**

**
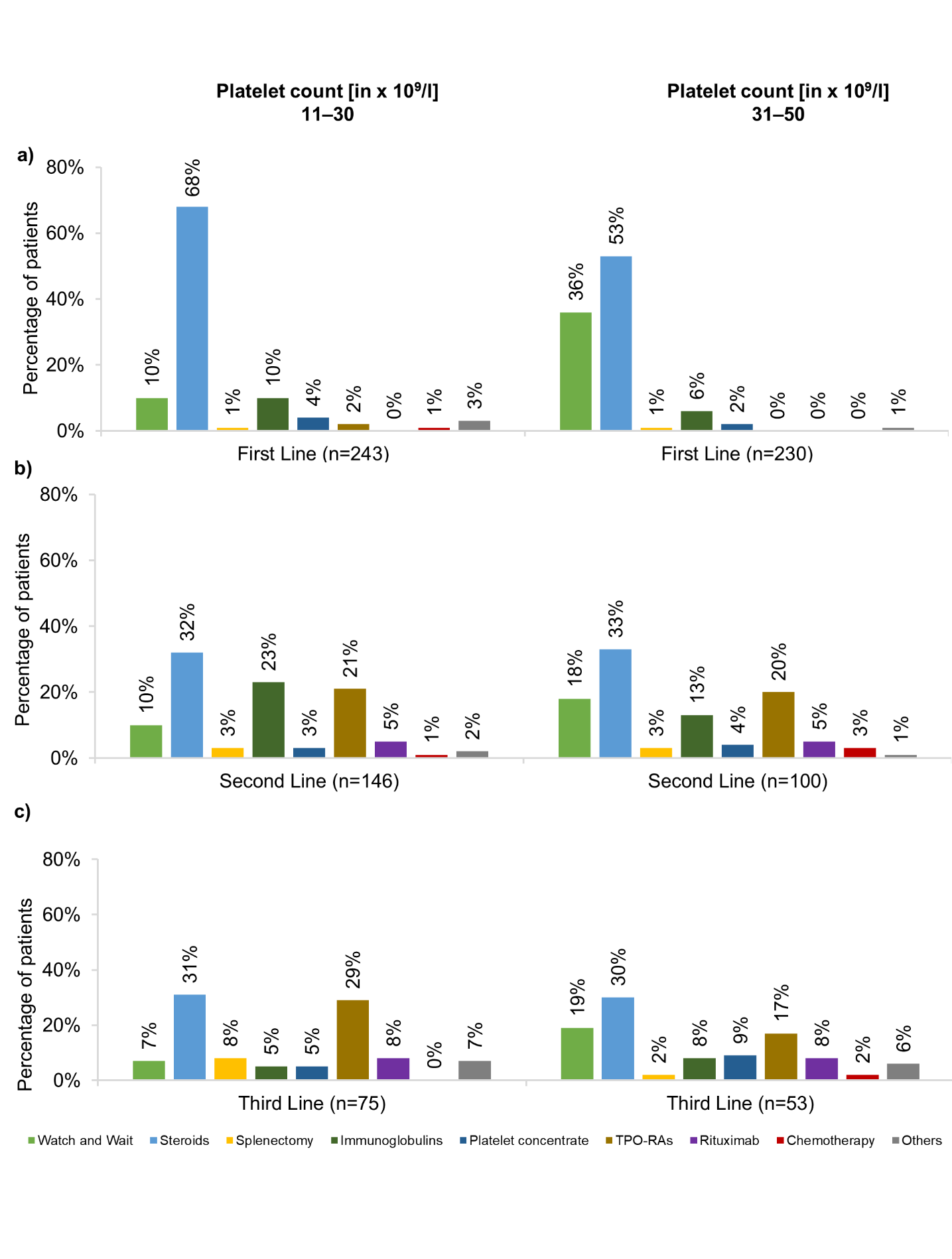
**

**Supplemental Fig. 3 Treatment strategies according to platelet count during a) first-, b) second- and c) third-line treatment. Multiple answers allowed (in this case n equals the number of answers given and not number of patients; percentages indicate main answer given and not the proportion of patients receiving treatment).**

**
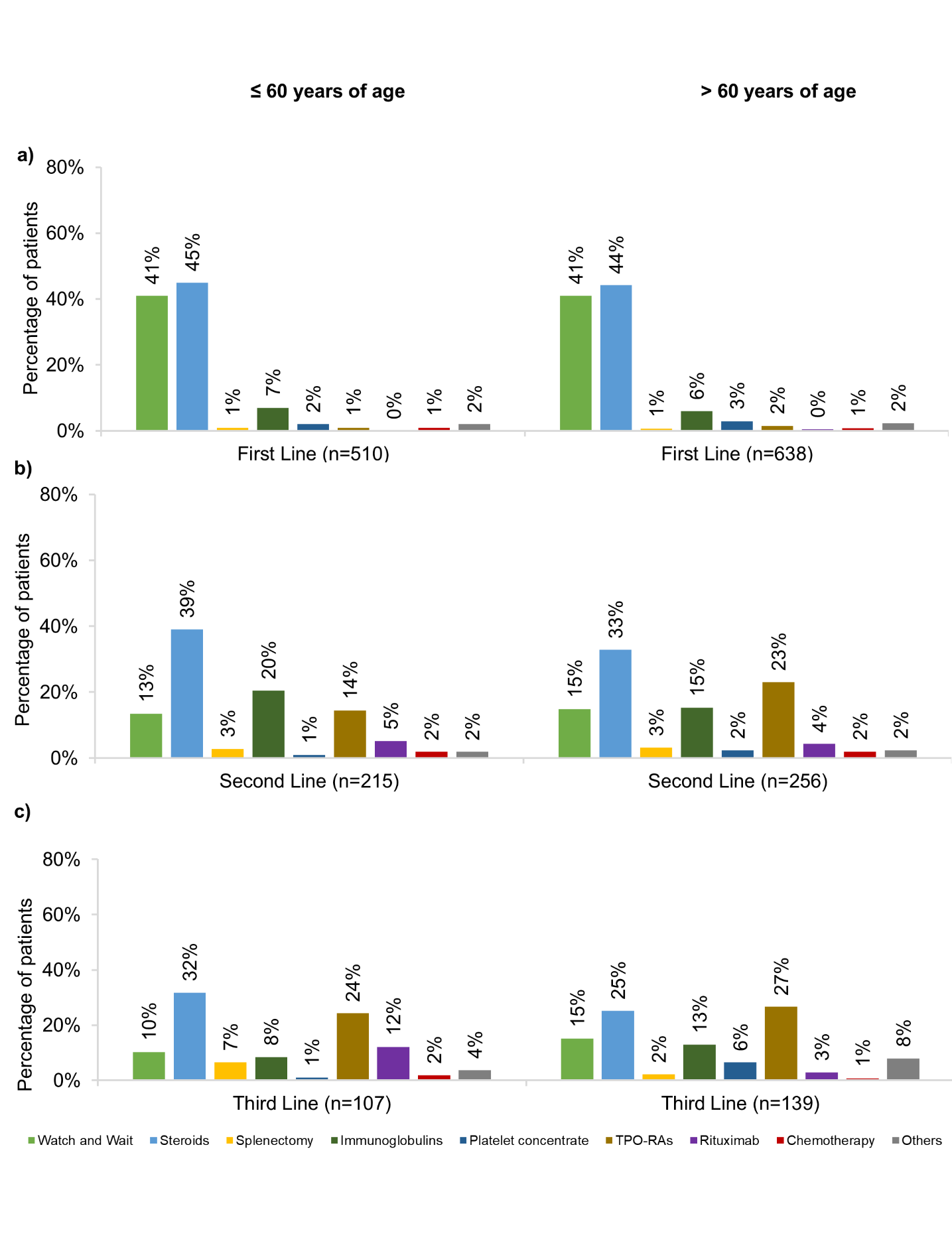
Supplemental Fig. 4 Treatment strategies according to patient’s age a) first-, b) second- and c) third-line treatment. Multiple answers allowed (in this case n equals the number of answers given and not number of patients; percentages indicate main answer given and not the proportion of patients receiving treatment).**
